# Effect of curcumin on the modulation of αA- and αB-crystallin and heat shock protein 70 in selenium-induced cataractogenesis in Wistar rat pups

**Published:** 2011-02-04

**Authors:** R. Manikandan, M. Beulaja, R. Thiagarajan, M. Arumugam

**Affiliations:** 1 Department of Animal Health and Management, Alagappa University, Karaikudi, India; 2 Department of Zoology, University of Madras, Guindy Campus, Chennai, India; 3 Department of Biotechnology, School of Chemical and Biotechnology, SASTRA University, Thanjavur, India

## Abstract

**Purpose:**

To investigate the expression of αA- and αB-crystallin and heat shock protein 70 (Hsp 70) during curcumin treatment of selenium-induced cataractogenesis in Wistar rat pups.

**Methods:**

Group I Wistar rat pups received only saline and served as the control. Group II Wistar rat pups were intraperitoneally injected with selenium (15 µM/kg bodyweight) to induce cataract. Group III Wistar rat pups also underwent selenium-induced cataract but were cotreated with 75 mg/kg body weight of curcumin (single oral dose). Group IV Wistar rat pups with selenium-induced cataract were post-treated with curcumin at the group III dosage 24 h after selenium administration. Group V Wistar rat pups with selenium-induced cataract were pretreated with curcumin at the group III dosage 24 h before selenium administration.

**Results:**

This study found higher levels of αA- and αB-crystallin and Hsp 70 in lenses injected with selenium alone (group II) than in control lenses (group I). Similar results were observed in the group III and IV lenses. In contrast, in group V, the presence of curcumin 24 h before selenium injection decreased the αA- and αB-crystallin and Hsp 70 levels to almost the same as those found in group I lenses.

**Conclusions:**

Curcumin suppressed the expression of selenite-induced αA- and αB-crystallin and Hsp 70, and may therefore suppress cataract formation in rat pups.

## Introduction

Pathophysiologically, one of the early signs of cataractogenesis is damage to the lens cell membrane [1], apparently due to a decline in its ability to actively transport substances against electrochemical gradients, resulting in alterations of intraocular composition and metabolism. The connection of these early effects to subsequent opacification of the tissue, characterized by the presence of disulfide-linked proteins of higher molecular weights with lower solubility [2], is quite complex. Such crosslinks are primarily established between lens glutathione and proteins, especially lens crystallins.

Cataract results from loss of transparency of the normal crystallin eye lens, and it is the major cause of blindness. However, many risk factors are associated with the pathogenesis of cataract, among which aging, diabetes, are the dominant risk factors [3-5]. The eye lens contains a high concentration of α-crystallin, the predominant protein of the vertebrate eye lens, and constitutes up to 50% of the total water-soluble fraction. It is normally composed of two subunits, αA- and αB-crystallin, both with a molecular weight of approximately 20 kDa [6,7]. The α-crystallin, by virtue of its special structural features and high concentration, contributes to the flawless transmission of light to the retina, with minimal scatter and spherical aberration [6-8]. Moreover, the homo- and hetero-oligomers of αA- and αB-crystallins [8] are known to function like molecular chaperones [9] in suppressing the aggregation of proteins [10] and inactivation of enzymes due to heat [11], UV light [10], and chemical agents [12]. Furthermore, many studies corroborate the critical role of αA- and αB-crystallin’s chaperone-like function in the maintenance of lens transparency [6,7,13]. Both αA- and αB-crystallin belong to the small heat shock protein (sHsp) family and share approximately 57% sequence homology [8].

Hsp 70, a major member of the sHsp family, is expressed in lens under unstressed conditions [14-15], suggesting that it is continuously needed for maintaining a normal lens microenvironment [16-17]. Further studies indicated that inducible Hsp 70, Hsp 40, and Hsp 27 are expressed in the lens epithelial cells under heat shock, oxidative and osmotic stress [14,17,18], suggesting that certain Hsps may play a part in protecting lens epithelial cells against a variety of stimulants that can damage or denature cell proteins. Apart from aging, various factors, such as nutritional deficiencies or inadequacies, diabetes, sunlight, environmental factors, smoking, and lack of antioxidants are known to increase the risk of cataract due to changes in Hsps [4,19].

Hence, the chaperone function of α-crystallin under hyperglycemic conditions is of great concern with respect to lens transparency. Indeed, α-crystallin from diabetic rat and human lenses has shown a substantial loss in its chaperone function [20,21]. Furthermore, α-crystallin chaperone activity was also found to be impaired in galactosemic rat lenses [22]. These studies imply that the impaired chaperone function of α-crystallin could be involved in the pathogenesis of selenium-induced cataractogenesis. We have previously shown that curcumin, the active principle of turmeric and a dietary antioxidant, scavenges free radicals and delays cataract in rat pups induced by selenium administration [23,24]. Therefore, in the present study, we have investigated whether curcumin modulates the chaperone activity of α-crystallin in selenium-induced cataractogenesis in Wistar rat pups.

## Methods

### Experimental animals

Male albino Wistar rat pups aged 8–10 days (d) weighing 15–20 g were procured from the National Institute of Nutrition, Hyderabad, India. All the experiments were approved by India’s Institutional Animal Ethical Committee guidelines (IAEC No. 360/01/a/CPCSEA). Rat pups were housed in an air-conditioned room at 22±2 °C with a 12 h:12 h light-dark cycle. Rat pups were fed with a balanced commercial rat diet (Hindustan Lever, Mumbai, India) and water ad libitum.

A pilot study was performed to determine the LD_50_ value for selenium (sodium selenite) in rat pups following the method of [25]. Briefly, selenium (10, 15, 20, 40, and 60 μM/kg body weight) was injected intraperitoneally into 8–10 d old suckling rat pups to determine the LD_50_, and both mortality as well as cataract formation were monitored. Selenium at a concentration of 60 μM/kg body weight (equivalent to 0.052 mg/kg body weight) when administered to 6 rat pups, resulted in mortality of 5 rat pups and formation of cataract in 1 rat pup. When the concentration of selenium was reduced, the mortality of rat pups decreased whereas cataract formation increased. The presence of cataract was determined by the formation of opacity, which was visible to the naked eye as yellowish white mass in the center of the lens. Thus, at a concentration of 15 μM/kg body weight (equivalent to 0.013 mg/kg body weight) injected to 6 animals, resulted in mortality of 3 rat pups and formation of cataract in 3 rat pups. The above results were observed after 3 to 4 d of selenium exposure. Thus, this dose was subsequently used in all further experiments. Similarly, curcumin was initially tested at various concentrations (25, 50, 75, 100, and 125 mg/kg body weight) by analyzing cataract inhibition. Curcumin at a concentration of 75 mg/kg body weight and above alone could prevent cataractogenesis, and thus, the lowest effective dose was selected. Curcumin at 25 and 50 mg/kg body weight could not prevent cataractogenesis.

The rat pups were divided into five groups (six in each group):

Group I: Control rat pups administered physiologic saline.
Group II: Selenium-induced pups (15 µM/kg bodyweight; single dose of selenium).
Group III: Selenium-induced pups cotreated with curcumin (single oral dose of curcumin at 75 mg/kg bodyweight)
Group IV: Selenium-induced pups post-treated with curcumin (after 24 h; group III dosage).
Group V: Selenium-induced pups pretreated with curcumin (group III dosage) 24 h before the administration of selenium.

### Isolation of lenses

After treatment, rat pups were sacrificed by means of an overdose of pentobarbital at a dose of 50 mg/kg body weight, given intraperitoneally, and the eyes were enucleated. Eye lens tissue was immediately dissected out, washed in ice-cold saline to remove blood, and frozen at -70 °C. The tissue homogenate of thawed eye lens was prepared using 0.1 M Tris-HCl buffer (pH 7.4), and the supernatants obtained after centrifugation (4,473× g; 30 min; 4 °C) were used for analysis of αA- and αB-crystallin and Hsp 70 protein expression.

### Immunohistochemical analysis

Immunohistochemistry was performed by the method of [26] on 5 µm paraffin-embedded tissue section on poly-L-lysine coated glass slides. The tissue sections were deparaffinized by placing the slides in an oven at 60 °C for 10 min and then rinsed twice in xylene for 10 min each. The slides were then hydrated in a graded ethanol series (100%, 90%, 70%, 50%, 30%) for 10 min each and then given a final rinse in double-distilled water for 10 min. The sections were incubated with 1% H_2_O_2_ in double-distilled water for 15 min at 22 °C, to quench endogenous peroxidase activity. Then the sections were rinsed with Tris-HCl containing 150 mM NaCl (pH 7.4) and blocked in blocking buffer (TBS, 0.05% Tween 20, 5% NFDM) for 1 h at 22 °C. After washing with TBS containing 0.05% Tween 20, the sections were incubated with the primary antibody, αA- and αB-crystallin polyclonal anti-rabbit IgG antibody (BD Biosciences, San Jose, CA), at a dilution of 1:500 overnight at 4 °C. After incubation, the eye lens sections were rinsed twice with TBS containing 0.05% Tween 20 and incubated with the secondary antibody, goat anti-rabbit IgG-HRP conjugate, at a dilution of 1:3,000 for 1 h at 4 °C. After washing with TBS containing 0.05% Tween 20, immunoreactivity was developed with 0.05% DAB and 0.01% H_2_O_2_ for 1–3 min, and the eye lens sections were microscopically (5×; Carl Zeiss Axioscop; Carl Zeiss, Berlin, Germany) observed under the bright field for brown color formation.

### Western blot analysis

The whole lenses from each age group were homogenized in 10 volumes (v/w) of 20 mM Tris-HCl (pH 7.4) containing 5 mM EDTA and 10 mM mercaptoethanol using a glass-glass dounce homogenizer. The homogenates were centrifuged at 6,440× g, 30 min, 4 °C and the recovered supernatants were used as lens protein. The lens proteins prepared from normal and treated rat pups were electrophoresed by the method of [27] in 12% SDS-PAGE slab gels. After electrophoretic separation, the proteins were transferred onto immubilon nitrocellulose membranes. Immunoblotting was performed with primary antibody (αA- and αB-crystallin polyclonal anti-rabbit IgG antibody, 1:3,000 dilution) after blocking with nonfat dry milk powder (NFDM), and later incubated with peroxidase-tagged goat antirabbit IgG antibody. Immune complexes were detected using diaminobenzidine (0.01%) and H_2_O_2_. Quantification of band intensity was done using a gel doc system using Quantity One software (version 4.0; Bio-Rad, Hercules, CA).

### Reverse-transcription polymerase chain reaction

Total RNA was extracted from normal and cataractous lenses by the acid guanidium thiocyanate-phenol-chloroform extraction method [27] using Trizol reagent (Sigma St. Louis, MO) according to the manufacturer’s instructions (TECHNE, Techgene, UK). Oligo-dT-primed first stand cDNA was prepared from total lens RNA using AMV reverse transcriptase at 37 °C for 60 min. PCR was performed with gene-specific primers using Taq DNA polymerase (Bangalore Genei, Bangalore, India). The primers used for αA- and αB-crystallin and Hsp 70 were based on the rat sequences 5′-CAC CGT GAA GGT ACT GGA AG-3′ (sense), 5′- TCA GGA AGG CAG ACT CTT G-3′ (antisense), 5′-AGA GCA CCT GTT GGA GTC TG-3′ (sense), 5′-TTC CTT GGT CCA TTC ACA GT-3′ (antisense), 5′-ATG AAG GAG ATC GCC GAG G-3′ (sense), and 5′-GTC GAA GAT GAG CAC GTT G-3′ (antisense), respectively. The following cycling conditions were used: 120 s of initial denaturation at 94 °C followed by 30 cycles of 90 s at 94 °C, 60 s at 60 °C, and 60 s at 72 °C, followed by 5 min at 72 °C. The primers used for β-actin (*Actb*) were 5′-GTG GCC GCT CTA GGC ACC A-3′ (sense) and 5′-CGG TTG GCC TTA GGG TTC AGG GGG G-3′ (antisense). The amplification products were electrophoresed on agarose gel (2%) in Tris-acetate EDTA buffer (pH 8.2). Bands stained by ethidium bromide were visualized by a UV transilluminator (Bio-Rad gel doc system) and the band intensity was analyzed densitometrically using Quantity One software (version 4.0; Bio-Rad).

## Results

### Effects of curcumin on eye lens αA- and αB-crystallin expression

#### Immunohistochemical analysis

**Figure 1:**
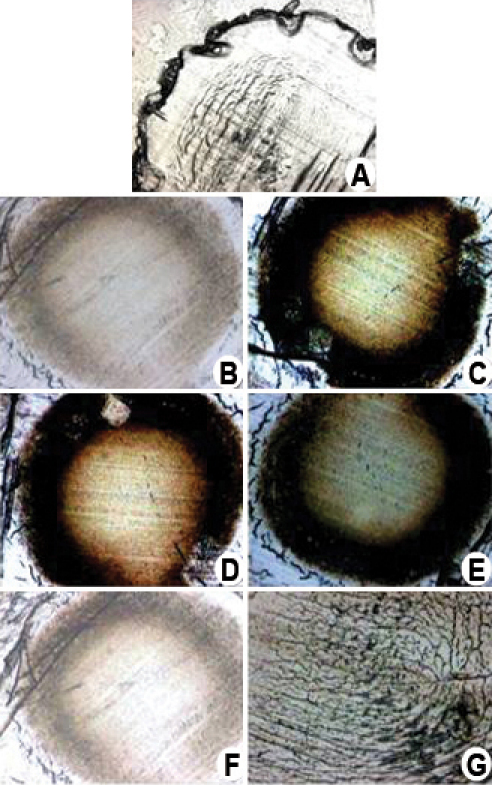
Effects of curcumin on immunohistochemistry of αA- and αB-crystallin in the eye lens of Wistar rat pups exposed to selenium. **A**: This image shows the control lens incubated in saline alone without any antibody treatment. **B**: This is a control lens that was incubated with saline and subjected to antibody treatment. **C**: Lens from rat pups administered with selenium alone. **D**: Lens from rat pups administered with selenium and curcumin simultaneously. **E**: Lens from rat pups administered with selenium first and then treated with curcumin after 24 h. **F**: Lens from rat pups pretreated with curcumin and then administered with selenium after 24 h. Lens sections were preincubated with αA- and αB-crystallin polyclonal antirabbit Immunoglobulin G (IgG) antibody (1:3,000 dilution) and subsequently with goat anti-rabbit IgG-horse radish peroxidase (HRP) conjugate (1:3,000 dilution). The immunoreactivity was developed with 0.01% 3,3-diaminobenzidine tetrahydrochloride (DAB) and H_2_O_2_. Note the brown color formation indicative of peroxidase reaction in the nucleus. Image **G**, negative control, lens treated with goat anti-rabbit IgG-HRP and developed using DAB and hydrogen peroxide (H_2_O_2_). The figure shows the high level of αA- and αB-crystallin expression induced by selenium-mediated oxidative stress.  This increased crystallin expression and aggregate formation was prevented by curcumin pretreatment.

Immunohistochemical localization of αA- and αB-crystallin in eye lenses performed on animals injected with selenium alone (group II; Figure 1C) revealed a higher αA- and αB-crystallin staining compared to the control (group I; Figure 1B). Similar results were also observed in lenses of group III (Figure 1D) and IV (Figure 1E) animals. However, in the case of group V (Figure 1F) animals, the presence of curcumin 24 h before selenium injection led to a decrease in αA- and αB-crystallin staining similar to that of group I.

#### Western blot analysis

**Figure 2:**
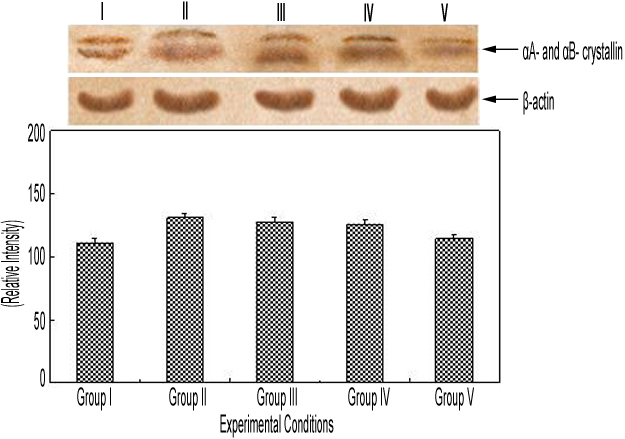
Immunoblot expression of αA- and αB-crystallin in control and experimental group of animals. Lane I, eye lens protein from control (physiologic saline) rat pups (group I); lane II, eye lens protein from selenium-injected rat pups (group II); lane III, eye lens protein from rat pups administered selenium and curcumin simultaneously (group III); lane IV, eye lens protein from rat pups injected with selenium 24 h before being administered with curcumin (group IV); and lane V, eye lens protein from rat pups administered with curcumin 24 h before being injected with selenium (group V). The separated lens protein was preincubated with αA- and αB-crystallin polyclonal antirabbit IgG antibody (1:3,000 dilution) and subsequently with goat antirabbit IgG-HRP (1:3,000 dilution). The immunoreactivity was developed with 0.01% DAB and H_2_O_2_. β-Actin refers to house keeping protein expression and its levels are constant across all treatment groups indicating the normal behaviour of lenses under various treatment. The figure clearly shows increased αA- and αB-crystallin protein expression under selenium-mediated oxidative stress. This increased crystallin protein expression was prevented by curcumin pretreatment.

Eye lens homogenate supernatant harvested from the rat pups and maintained in Tris-HCl buffer were subjected to SDS-PAGE (12%) under nonreducing conditions and processed for western blot analysis using αA- and αB-crystallin antirabbit IgG antibody. This was performed to detect the αA- and αB-crystallin in the lenses of animals injected with selenium alone (group II). This revealed higher levels of both crystallins when compared to the control (group I; Figure 2). Similar results were also observed in the lenses of group III and IV animals. However, in the case of group V animals, the presence of curcumin 24 h before selenium injection led to a decrease in the levels of both crystallins, and these were similar to the control (group I) level. Western blot analysis of eye lenses detected a double, clear, prominent fraction at the proximal part of the membrane with an estimated molecular weight of approximately 20 kDa.

### Reverse-transcriptase PCR analysis of αA- and αB-crystallin and heat shock protein 70 in the eye lens

**Figure 3:**
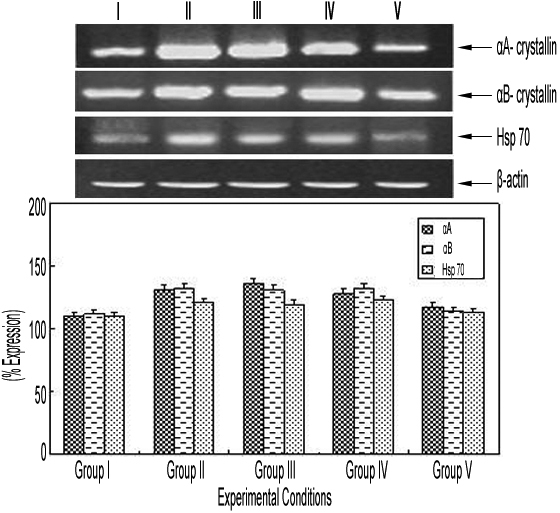
Effect of curcumin on αA- and αB-crystallin and heat shock protein 70 gene expression in the eye lens of Wistar rat pups exposed to selenium. Lane I, mRNA expression in lens from rat pups treated with saline alone (group I); lane II, mRNA expression in lens from rat pups administered with selenium alone (group II); lane III, mRNA expression in lens from rat pups administered with selenium and curcumin simultaneously (group III); lane IV, mRNA expression in lens from rat pups administered with selenium first and then treated with curcumin after 24 h (group IV); and lane V, mRNA expression in lens from rat pups pretreated with curcumin 24 h before selenium administration. Selenium-mediated oxidative stress causes an increase in the expression of αA- and αB-crystallin and Hsp 70 gene, which probably underlies the pathogenesis of cataractogenesis induced by selenium. All these changes were prevented by curcumin pretreatment, indicating its protective effect.

The relative percentage of expression of candidate genes in selenium-induced cataractogenesis was analyzed by the expression of β-actin in control animals and was found to be 100%. The αA- and αB-crystallin and Hsp 70 expression in the eye lenses was found to be elevated in group II (Lane II) animals, as compared to the control (group I; Lane I; Figure 3). A similar increase in αA- and αB-crystallin and Hsp 70 expression was also observed in group III (Lane III) and group IV (Lane IV) animals when compared to group I lenses. Interestingly, in the animals administered with curcumin 24 h before selenium injection (group V; Lane V), the expression was observed to be decreased to a level almost equal to that of the control (Figure 3).

## Discussion

Cataract is a major cause of blindness in an estimated 20 million people worldwide, especially in developing countries. Surgery is the only effective treatment for cataract, since the exact mechanism for its formation is still not sufficiently clear. Although cataract surgery is recognized as being one of the safest operations, there is a significant rate of complications, leading to irreversibly blind eyes. Pharmacological interventions to inhibit or delay lens opacification are still at the experimental stage. Studies are being conducted to explore the mechanism of cataractogenesis using various models of cataract and to target crucial steps to halt this process. Limitations in acceptability, accessibility, and affordability of cataract surgical services make it more relevant and important to look into alternative pharmacological measures for treatment of this disorder [28]. Thus, much enthusiasm is directed toward identifying natural compounds that will help to prevent cataractogenesis.

In addition to the calpain-mediated opacification of eye lens proteins [29], one group of protein that is significantly affected by oxidative stress is the α-crystallins, which are structural constituents of the eye lens. It is known that in eye lenses the α-crystallin exists in at least two forms, namely αA- and αB-crystallin. Among these, the distribution of αA-crystallin is restricted to the eye lens and αB-crystallin is ubiquitously present. Furthermore, α-crystallin is clearly needed for maintaining lens transparency, and in maintaining the solubility of αB-crystallin in the lens [6]. Interestingly, the ratio of αA- and αB-crystallin found in the eye lens is 3:1, and any change in their ratio appears due to pathology in the eye lens [7,13]. Due to their slow turnover, eye lens damage accumulates over time, subsequently leading to improper transmission of light by the lens. In the present study, the expression of both αA- and αB-crystallin increased in the eye lenses of animals when they were subjected to selenium exposure. Administration of curcumin either simultaneously or 24 h after selenium injection failed to produce any change in the mRNA expression of these proteins. This probably indicates that αA- and αB-crystallin levels are elevated as a cellular response to selenium-mediated stress. It is well established that crystallin levels are elevated in several age-related degeneration diseases wherein oxidative stress is implicated as a major event in their pathogenesis [30,31]. Thus, in the present study, the elevated mRNA expression of αA- and αB-crystallin upon selenium exposure, as well as αA- and αB-crystallin presence in tissues with high oxidative potential [32-36], clearly suggest that the expression of this gene is related to selenium-mediated stress.

Further, we have also investigated whether curcumin can amend the elevated expression of both αA- and αB-crystallin in the eye lens of rat pups exposed to selenium. Interestingly, administration of curcumin 24 h before selenium injection led to a decrease in the expression of both αA- and αB-crystallin in the eye lens. Our previous studies have shown that curcumin prevents antioxidant- and free radical-mediated cataractogenesis [23,24]. The present study strongly supports our contention that enhanced crystallin expression is linked to selenium-induced oxidative stress. Increased expression of α-crystallin might be a very important and vital cellular (lens) adaptation as a protective mechanism against environmental and/or metabolic stress, as has also been reported by [27].

The heat shock response is one possible protective mechanism to maintain a normal microenvironment in the lens. The lens is a closed system with a limited ability to repair or regenerate itself [37]. However, the eye chamber is constantly exposed to aqueous humor containing reactive oxygen species generated by light-catalyzed reactions [38]. These free radicals may cause increased oxidation in tissue of the anterior eye segment and finally lead to oxidative damage. Therefore, constitutive expression of Hsp 70 in the lens may be due to continual oxidative stress. Our results demonstrate that curcumin suppresses Hsp 70 expression in the eye lenses of rat pups exposed to selenium. Interestingly, administering curcumin 24 h before selenium injection has this important effect in the expression of Hsp 70 in the eye lens.

In summary, our study clearly demonstrated that the prior presence of curcumin is a prerequisite for it to function as a potent cytoprotective agent in preventing cataract development in the first place. Furthermore, this study clearly shows that regular dietary intake of curcumin would greatly reduce and prevent the occurrence of cataractogenesis in Wistar rats. In conclusion, this study shows the cytoprotective nature and novel mechanism of action of curcumin in preventing selenium-induced cataractogenesis by inhibiting the expression of αA- and αB-crystallin and Hsp 70 in eye lenses.
